# The perspectives of mapping and monitoring of the sense of self in neurosurgical patients

**DOI:** 10.1007/s00701-021-04778-3

**Published:** 2021-03-08

**Authors:** Karl Schaller, Giannina Rita Iannotti, Pavo Orepic, Sophie Betka, Julien Haemmerli, Colette Boex, Sixto Alcoba-Banqueri, Dorian F. A. Garin, Bruno Herbelin, Hyeong-Dong Park, Christoph M. Michel, Olaf Blanke

**Affiliations:** 1 Department of Neurosurgery, Geneva University Medical Center & Faculty of Medicine, University of Geneva, Rue Gabrielle-Perret-Gentil 4, 1205 Geneva, Switzerland; 2 Functional Brain Mapping Laboratory, Department of Fundamental Neurosciences, University Geneva, Geneva, Switzerland; 3 Laboratory of Neurocognitive Science, Center for Neuroprosthetics and Brain Mind Institute, Swiss Federal Institute of Technology (EPFL), Geneva, Switzerland; 4 Department of Clinical Neurosciences, Geneva University Medical Center & Faculty of Medicine, University of Geneva, Geneva, Switzerland

**Keywords:** Self, Consciousness, Social, Brain surgery, Neuropsychology, Bodily self

## Abstract

Surgical treatment of tumors, epileptic foci or of vascular origin, requires a detailed individual pre-surgical workup and intra-operative surveillance of brain functions to minimize the risk of post-surgical neurological deficits and decline of quality of life. Most attention is attributed to language, motor functions, and perception. However, higher cognitive functions such as social cognition, personality, and the sense of self may be affected by brain surgery. To date, the precise localization and the network patterns of brain regions involved in such functions are not yet fully understood, making the assessment of risks of related post-surgical deficits difficult. It is in the interest of neurosurgeons to understand with which neural systems related to selfhood and personality they are interfering during surgery. Recent neuroscience research using virtual reality and clinical observations suggest that the insular cortex, medial prefrontal cortex, and temporo-parietal junction are important components of a neural system dedicated to self-consciousness based on multisensory bodily processing, including exteroceptive and interoceptive cues (bodily self-consciousness (BSC)). Here, we argue that combined extra- and intra-operative approaches using targeted cognitive testing, functional imaging and EEG, virtual reality, combined with multisensory stimulations, may contribute to the assessment of the BSC and related cognitive aspects. Although the usefulness of particular biomarkers, such as cardiac and respiratory signals linked to virtual reality, and of heartbeat evoked potentials as a surrogate marker for intactness of multisensory integration for intra-operative monitoring has to be proved, systemic and automatized testing of BSC in neurosurgical patients will improve future surgical outcome.

## Introduction

What do neurosurgeons answer to patients, who are supposed to undergo elective intracranial procedures and who are asking “Doc, will I be the same person after surgery?” The traditional concerns of the neurosurgical community of course take into account potential post-operative personality changes in their patients, but compared to established feedback based on neuroscientific data in language, movement, and perception, feedback about potential changes to personality and the sense of self is not yet established. This is the case despite the widespread and important role of neuropsychologists in patient evaluation and increasing research on the sense of self. During consultations and consentment conversations, the issues of language or motor function, or of cranial nerve function, respectively, are regularly aborded. Usually, we come closest to the subject of *personality*, when we are explaining the various elements of language and speech, of receptive and expressive language centers, and the respective cortical and subcortical structures involved in its organization. Awake craniotomy may be suggested in order to peri-operatively assess language functions. The examination and mapping of *personality* and sense of self is not discussed on a routine basis, however, and not yet based on neuroscience data. The frequency of post-operative deficits of the sense of the self is difficult to quantify, as modern post-operative clinical neuropsychological evaluation does not incorporate specific dedicated tests of the sense of the bodily and the cognitive self. However, the authors are convinced that these deficits are underestimated in the post-operative period and may affect dramatically the social life and the working capacity of neurosurgical patients.

It is the goal of this perspective article to highlight the present state of knowledge about the underlying networks of brain structures contributing to a person’s sense of self and what deems possible to be added to the portfolio of pre-surgical evaluation and intra-operative surveillance of functions related to personality and of the human sense of self.

## Self-consciousness

Self-consciousness is one of the most astonishing features of the human mind and has been approached in diverse fields such as philosophy, psychology, psychiatry, and more recently in cognitive neuroscience. However, self-consciousness is notoriously difficult to define, encompassing emotional, spatial self, verbal, conceptual, and social aspects among others [71, 83]. William James was one of the first who differentiated two critical notions of self: a so-called physical self and a mental self [65], and several current cognitive neuroscience approaches to the sense of self often keep a related dichotomy and target two distinct and separated notions of self (i.e., [39, 44, 50]). Here, we juxtapose a bodily self, based on mainly perceptual multisensory and sensorimotor mechanisms, with a cognitive self, based on language, memory, and other cognitive functions (i.e., [39, 44, 50]).

### Bodily self and bodily self-consciousness

On the one hand, researchers have investigated a fundamental aspect of the self and its link with bodily perception: the bodily self. This implicit and pre-reflexive experience of being the subject of a given experience is proposed to be a low-level self-representation, based on perceptual mechanisms of multisensory bodily signals [83]. The term bodily self-consciousness (BSC) has recently been proposed [20, 47], and core BSC features are self-identification (i.e., the degree to which an organism identifies with the content of a global body representation), body ownership (i.e., the feeling that the physical body and its parts, such as its hands and feet, belong to “me” and are “my” body), self-location (i.e., a determinate volume in space, normally localized within the bodily boundaries as represented), first-person perspective (i.e., the experience of the position from where “I” experience to perceive the world), and the sense of agency (i.e., the feeling of being in control of one’s own actions) [15, 17, 18].

BSC research has investigated brain mechanisms that process body-related sensory signals originating from the space outside the body (i.e., exteroceptive bodily signals). However, the brain also receives other bodily, interoceptive, signals from the inner organs (i.e., cardiac, respiratory, and intestinal). Importantly, several authors have made an influential argument that the interoceptive brain mechanisms play a fundamental role in BSC [8, 11, 87]. Based on philosophical intuition [32, 33] and indirect evidence [8, 25, 34], this proposal for a neural BSC system based on interoceptive signals (i-BSC [87]) was developed by Antonio Damasio and AD Craig and has recently received experimental support [36, 38, 40, 41, 43]. Our own work was directly inspired by these proposals and driven by the ambition to investigate whether exteroceptive and interoceptive bodily signals contribute to BSC. For this, we developed a series of virtual reality setups that allowed to expose participants to multisensory stimulation including interoceptive as well as exteroceptive cues, revealing that this type of stimulation also allowed to manipulate BSC [1, 3, 7, 15, 87, 92]. Moreover, visual stimuli, which are presented synchronously or asynchronously with the frequency of the individual’s heartbeat, are interesting stimuli to investigate awareness [106]. Importantly, these findings suggest that BSC should be conceived as an integrated single system (x-BSC [87]), based on exteroceptive and interoceptive bodily signals. Merging behavioral, neuroimaging, neurological, and electrophysiological evidence, the x-BSC system has been proposed to reconcile the two largely separated views in terms of interoceptive and exteroceptive bodily contributions to BSC (for an extensive proposal why and how the integration between interoceptive and exteroceptive information is thought to be important for the sense of self, see the recent work from Park and Blanke [87]).

### Cognitive self

The second notion of the self includes the many cognitive processes that are associated with the self (i.e., the cognitive self) [83, 99]. As opposed to the pre-reflective and perceptual lower-level bodily aspects of the bodily self, the cognitive self is rather of a reflective and integrated nature and involves cognitive functions such as language, memory, mental imagery, and symbolic and abstract aspects [83]. In other words, the cognitive self regroups higher-order cognitive processes allowing a subject to reflect and understand whether a specific mental content is related to themselves and their own person. Different cognitive components related to the cognitive self can be investigated such as the recognition of self-related cues, i.e., the visual recognition of one’s own face [129] or the auditory recognition of one’s own name or one’s own voice [12, 94, 124]. This also includes memory components such as the recall of personally relevant information, or of one’s own personality, by assessing episodic and autobiographical memories [25, 26, 53, 102]. The cognitive self, as defined here, overlaps with previous notions of such higher-level notions of self, such as James’ mental self, Damasio’s extended self [39, 65], or Gallagher’s [50] and Dennett’s narrative self [44]. It should also be noted that, although the conceptual distinction and neural mechanisms are distinct between bodily and cognitive self, both notions overlap. For example, multisensory stimulations have been shown to not only impact the bodily self, but also the cognitive self, including episodic memory [25], language processing [27], and spatial navigation [78]. Accordingly, we have recently shown that viewing the body during the encoding of scenes in VR facilitates later episodic memory for these events and impacts the neural correlates [25]. Moreover, an enhanced state of BSC for a self-avatar was found to improve spatial navigation performance and to alter activity in the medial temporal lobe [78]. During a body swap illusion, recent work showed flexibility of the self-concept in function of the strength of the illusory body ownership; such illusion was also modulating episodic memory [125]. The self, like many representations in humans, requires constant sub- or unconscious monitoring of the physical and the physiological state, i.e., by neural processing of incoming interoceptive (signals from the heart, respiration, and from viscera) signals and to match them with exteroceptive (i.e., audio-visual and tactile) data [21]. As the integrative target organ is the brain, in accordance with present-date concepts, neurosurgeons should be familiar with basic principles of the construction of the self and take potential impact of intracranial procedures on the self of their patients into consideration.

## Experimental BSC paradigms

One approach to test the integrity of BSC is to investigate the perception of multisensory bodily stimuli by exposing people to conflicting sensorimotor inputs using different techniques (e.g., VR) and experimental paradigms. To highlight the importance that the multisensory integration processes are thought to play in BSC, we are describing below two classical paradigms.

### The rubber hand illusion

In the so-called rubber hand illusion, the experimenter strokes a fake hand by hand or with a stick or another object simultaneously with the participant’s own yet hidden hand. This application of visuo-tactile stimulation is carried out for some time (1–2 min or longer) and leads to alterations of BSC, especially illusory touch and hand ownership for the seen hand. Such BSC changes are decreased or not observed when the visuo-tactile stimulations are asynchronous, when the orientation in space of the participant’s hand and the fake hand is misaligned, or when a control object is stroked (i.e., a box instead of a fake hand), suggesting the importance of multisensory integration in such phenomenon [22, 119]. These alterations in BSC are evaluated by using questionnaires, by “proprioceptive drift” measures (i.e., participants perceive their hand to be at a position that is displaced towards the fake hand [24]), skin conductance response changes [4], and multisensory measures such as cross-modal congruency effects [6] and peripersonal space measures [82]. The RHI paradigm has also been developed using VR [109, 127] and also allowed online manipulation of interoceptive feedback using cardio-visual stimulation (i.e., Suzuki et al. found that illusory ownership of a virtual hand is observed if a virtual hand is illuminated synchronously with respect to the participant’s online detected cardiac signals [123]).

### The full-body illusion

The full-body illusion (FBI) paradigm generalizes principles from the rubber hand illusion and applies them to the entire body, usually using mixed reality. The test person is wearing a VR helmet with a head-mounted display, through which a virtual avatar of that very same person can be seen from the back and as projected at a distance of 2 m in front of the participant [72]. Then, the experimenter applies tactile stimulations on the subject’s back and such stimulations will be displayed to the participant in the VR helmet and as seen online on the back of the avatar in VR. This is done either synchronously or asynchronously (with an additional 500-ms delay), as in the rubber hand illusion paradigm. The synchronous condition gives rise to changes in BSC, including illusory self-identification, illusory self-location over the virtual body [6, 47, 72, 95], and changes in the first-person perspective [63, 97]. These alterations in BSC are evaluated by using questionnaires, by drift in self-location using walking responses [72] (i.e., participants perceive their body to be at a position that is displaced towards the position of the avatar) or mental imagery responses [63], skin conductance response changes [47], and multisensory measures such as cross-modal congruency effects [6] and peripersonal space measures [82, 115]. Several variants of the full-body illusion exist [47], and online manipulation of interoceptive feedback using cardio-visual stimulation has also been used to induce changes in self-identification and self-location [7] (see the section “Bodily self: full-body illusion based on exteroceptive and interoceptive signals”). A similar paradigm using a first-person perspective rather than a third one has been developed and led to the perceptual illusion of body swapping [95]. These BSC paradigms are of clinical relevance and have been automatized and translated to several clinical applications, leading to novel neuroprosthetics chronic leg pain therapies for patients with neuropathic pain in spinal cord injury [98], complex regional pain syndrome [120], bionic limbs and peripheral nerve stimulation [103], and severe leg pain and spinal cord stimulation [121].

## Monitoring and mapping in neurosurgery

Direct cortical stimulation (DCS) via the application of electric currents, and stimulation of the cerebral white matter allow for functional allocation and for continuous peri-operative mapping and monitoring of the intactness of the concerned tracts and functions [45, 46, 59, 60, 84, 101, 105]. The introduction of phase reversal for the precise allocation of the central sulcus, in combination with transcranial or direct subsequent motor mapping and motor evoked potential (MEP) monitoring, has allowed to define entry zones for surgery in and around the central region and for the placement of subdural strip electrodes for continuous MEP monitoring during i.e. tumor surgery or during resective epilepsy surgical procedures in the vicinity of the somatosensory cortex [23, 28, 93]. Subsequent developments in monitoring and mapping of cerebral functions, of white matter fiber tracts, and cranial/peripheral nerves have since become a mainstay of functional preservation during neurosurgical procedures [29, 45, 46, 79, 81, 114]. Be it performed under general anesthesia, or during awake craniotomies, the development of these techniques has allowed to push the limit for resective intracranial (and intraspinal) procedures toward higher resection rates, prolonged progression-free survival (PFS), and better functional outcomes and quality of life (QOL) [43, 45, 80]. However, most attention is so far focused on brain regions controlling language, motor functions, and perception (i.e., mapping the so-called eloquent cortex) [29, 45]. Recent developments include the more routine use of monitoring of the visual pathways with visual evoked potentials (VEP) recording, olfactory monitoring, and the integration of stimulation-mapping tools into suction and resection devices [22, 58, 77, 101]. Language mapping under general anesthesia is under clinical evaluation still [73].

Finally, the Bispectral index™ (BIS) can be applied during neurosurgical procedures to monitor the hypnotic state of the patient during the surgery. It is based on various electroencephalogram (EEG) parameters recorded by the mean signals of scalp electrodes such as time and frequency domain components and returns a single BIS number [118]. This score represents the depth of anesthesia: the lower the score, the deeper the anesthesia. Typical values would be BIS > 97 for awake unsedated individuals, BIS = 60 for unconsciousness induced by drugs, BIS = 30 when EEG shows burst suppression, and BIS = 0 for a flat-line EEG. During intracranial surgeries, ideally, the BIS should range between 40 and 60. The online analyses of EEG are routinely performed using sensorimotor monitoring or visual evoked potentials, as aforementioned. The same type of analyses can be adapted to scrutinize residual cognitive cerebral functions like it has been done for measuring phonological processing of language [73] or analyses of epileptic discharges [128].

## Anatomical substrates for the bodily and the cognitive self

Despite the abovementioned developments, stimulation of and/or damage to the insular cortex (i.e., direct physical, or due to ischemic lesions) due to resection of insular pathologies has been reported to be associated with changes in personality and self, but also autonomous regulation, emotional, and sexual functions [2, 33]. Given the role of the insula and related structures in BSC, and when aiming at mapping and preserving related aspects of sense of self and personality, it is thus mandatory to investigate how the human insula and related structures in the context of multisensory bodily signals and the role of such processing in the patient’s self-consciousness and how it relates to the two main aspects of the sense of self, the bodily self (BSC) and the cognitive self [35, 87]. Whereas the latter is based on different cognitive systems such as recognition, language, memory, or mental imagery, the former is based on multisensory perception, including interoceptive and exteroceptive information. However, it is currently unknown whether the potential post-surgical mental changes in personality and sense of self relate to the bodily or cognitive self or to both. Pre- and peri-operative investigation of the bodily self and the cognitive self and especially their dependence on interoceptive bodily cues as mediated by the insular cortex, medial prefrontal cortex, and temporo-parietal junction (that all have been linked to self-related processing) would require the definition of measurable biomarkers, which are representative for various domains of the human self [41, 87]. The main neural relay structures for ascending interoceptive signals, i.e., from the viscera and glands, are located in the insula and in the cingulum [8, 87].

To maintain normal BSC, with normal self-identification with the body, self-location and first-person perspective multisensory perception and integration of exteroceptive and interoceptive information is important. Signals of such external or exteroceptive information may be recorded in a dedicated cortical network of multimodal neurons, spanning between the premotor cortex, the inter-parietal cortex, and the temporo-parietal junction [40, 57, 63, 64, 87, 96].

Interoceptive signals in BSC based on visceral afferents and their neural processing play a critical role in self-consciousness and traditionally relate to a different neural system [20, 31, 33, 36, 40, 88]. In particular, the insula and cingulate cortex have been proposed as primary cortical projection sites of interoceptive signals and critical for self-consciousness [34, 116]. Recent evidence supports these theoretical proposals [87, 106, 108]. For instance, Park and colleagues reported that spontaneous fluctuation of neural responses to heartbeats in the ventromedial prefrontal cortex (vmPFC), anterior cingulate cortex (ACC), and angular gyrus predict participants’ visual detection performance [89]. Moreover, they suggested that such neural events time-locked to heartbeats might carry information related to the subjective dimension of conscious visual experience and may relate to the bodily self [88]. More recent studies directly investigated the link between neural responses to heartbeats and the bodily self [7, 21, 90]. Neural responses to heartbeats in the posterior cingulate cortex and insula have been shown to covary with experimentally induced changes in self-identification [90], suggesting that bodily signals and their neural processing in cortical midline structures and insular cortex are associated with the bodily self. There are other sources of continuous interoceptive information, however, that is of relevance for BSC, such as the cyclic breathing signals [1, 3, 15] and gastric signals [8, 126]. However, it is currently still unclear, if this neural system of bodily self (BSC), involving exteroceptive and interoceptive signals [87], serves as sort of a scaffold for higher level functions involved in the cognitive self.

## Neural mechanisms of the bodily self

Recent cognitive neuroscience has aimed at revealing neural mechanisms of the bodily and cognitive self. A recent meta-analysis suggests that BSC recruits a network of multisensory brain areas located in the intraparietal sulcus (IPS) region, premotor cortex (PMC), and temporal-parietal junction (TPJ) [20, 25, 56]. These results are based on experimental manipulations of BSC using virtual reality (VR). For instance, tactile stimulation of a subject’s hand/body shown in immersive VR gives rise to illusory self-identification and illusory self-location over a virtual body (i.e., full-body illusion, FBI [6, 47, 72, 95]). In other recent work integrating fMRI with VR, synchronous visuo-tactile stroking of the real and virtual body, inducing illusory self-identification and self-location, was found to activate IPS, PMC, TPJ, and the putamen [56, 96]. Another VR-fMRI study found that TPJ activity (peaking in the posterior superior temporal gyri, the parietal operculum, and the posterior insula) was associated with changes in self-location and in the experienced direction of first-person perspective [63]. The involvement of the insula was recently confirmed by a 7T fMRI study [21] and by functional connectivity analysis [64] showing that the patterns of functional connectivity from the TPJ to the insula and medial prefrontal cortex reflected experimentally induced changes in self-location and first-person perspective.

Conscious access to interoceptive bodily signals has been proposed to inform the basis of selfhood [9, 37, 38, 116, 117]. One potential way to tackle the bodily self is to measure how accurately participants feel their internal body signals (i.e., signals coming from the glands and viscera), a measure also called interoceptive accuracy [51]. Different interoceptive channels can be objectively assessed (e.g., respiratory, rectal, or gastric), but the cardiac interoceptive channel (i.e., cardioception) is arguably the easiest to measure and, therefore, is a classic way to assess interoceptive processes [16, 51, 52]. The most popular tasks measuring cardioception are the heartbeat tracking and the heartbeat discrimination task [66, 110]. During the heartbeat tracking task, participants are asked to silently count their own heartbeats during a given period of time. Meanwhile, a pulse oximeter attached to their index finger (or ECG) tracks the participants’ actual heartbeat. The greater the overlap between the participant’s subjective heartbeat count and the objective recording, the higher the interoceptive accuracy [14, 52, 110]. In the heartbeat discrimination task, participants hear a series of sets of ten sounds, some of which are played synchronously with their heartbeat and others are played with a delay in their heartbeat. Participants are asked to detect whether or not the sounds were played in sync with their heartbeat and higher accuracy in this task suggests better cardiac interoception [14, 51, 66]. In the functional magnetic resonance imaging scanner (fMRI—using a modified version of the discrimination task), attention toward heartbeat classically elicits enhanced activity in the insula, sensorimotor, and cingulate cortices [38]. Moreover, the right anterior insula (AI) activity positively predicts subjects’ interoceptive accuracy on the task. In addition, right AI gray matter volume correlates with both interoceptive accuracy and sensibility, suggesting that this brain region supports a representation of body states, accessible to awareness and potentially contributing to the bodily self [38, 116].

The above anatomical regions are frequently subject to neurosurgical procedures, including the resection of intrinsic tumors, for epilepsy surgery, or during extra-axial approaches to the skull base and to vascular pathologies, with all of them carrying the risk of vascular damage to i.e. the insula or to the cingulum and others. Consideration of potential effects on bodily self, cognitive self, and personality when performing surgery in these regions thus seems not only important, but also possible, adapting the reviewed procedures to neurosurgical patient evaluations.

## Effects of surgical resection on personality and mood

Cognitive, perceptual, and motor functions in candidates for brain surgery are usually assessed during pre-operative neuropsychological testing and have proved instrumental to plan, to predict, and to minimize surgery-induced damage and loss of function [69]. Recent research findings and clinical observations suggest that damage of the insular cortex (i.e., in the context of vascular or tumoral pathologies, or due to surgery) leads both to autonomous dysfunctions (e.g., changes in heart rate variability, intermittent tachycardia, hyperventilation) and to alterations of the sense of self (e.g., changes in personality and or self-identification) [42, 104]. Moreover, patients with insular pathologies may exhibit pre- or post-surgical psychological side effects, including mood changes or lack of stress resistance and empathy, which may relate to alterations in the sense of self and which often have important consequences in their daily life (e.g., divorce, professional and familial changes) [30]. Adapted and comprehensive patient information should also include the risk of post-surgical perturbation of those cognitive functions that might influence social behavior, personality, and the self.

We argue that post-surgical disturbances of the sense of self and personality, although complex and difficult to measure behaviorally and clinically, can be estimated by evaluations of the bodily self and cognitive self. One such condition following insula resection or damage that has been linked to personality and the sense of self is depersonalization. Depersonalization refers to the recurring and prolonged sensations of being detached from one’s body, as if being an outside observer, often associated with feelings of loss of control over one’s own body, actions, or thoughts. More work is needed on the brain mechanisms of depersonalization, but it has been argued frequently that they are linked to those of bodily and/or cognitive self [61, 119].

## Assessment and mapping of the sense of self in neurosurgery

### Bodily self: full-body illusion based on exteroceptive and interoceptive signals

One approach to test the integrity of BSC is to investigate the perception of multisensory bodily stimuli by exposing people to conflicting inputs using VR or other devices and setups.

Several variations of the full-body illusion paradigm have been developed (see the section “The full-body illusion”); one variation also includes interoceptive signals. Hence, in this cardio-visual full-body illusion, synchronous flashing of the silhouette of the avatar was shown to the participant, based on his or her own online detected heartbeat (ECG) or the respiratory pattern (respiration belt). These interoceptive full-body illusions also allowed to manipulate BSC [1, 3, 7, 15, 87, 92]. Moreover, visual stimuli, which are presented synchronously or asynchronously with the frequency of the individual’s heartbeat, are interesting stimuli to investigate awareness [106]. Here, again, the insular cortex has been confirmed to play a crucial role in filtering and submitting intero- and exteroceptive signals to a hierarchy, which is related to self-awareness [106, 130]. An extra-operative study on neurosurgical patients with and without insular tumors showed clear differences between the patients and the control group [106]: in the group with (anterior) insular tumors—as opposed to patients with lobar gliomas and to controls—the previously shown effect of cardio-visual suppression of visual stimuli, which were presented in synchrony with the heartbeat, was abolished. These findings underline the importance of the insula for the combined processing of interoceptive and exteroceptive information—and that is of relevance for the constitution of self-consciousness.

### Cognitive self: self-other discrimination based on auditory and visual signals

Auditory signals involved in self-voice processing are processed via different cerebral networks and further differ whether they concern one’s own voice (= self-voice) or someone else’s voice (= other voice) [32, 54].

**Fig. 1:**
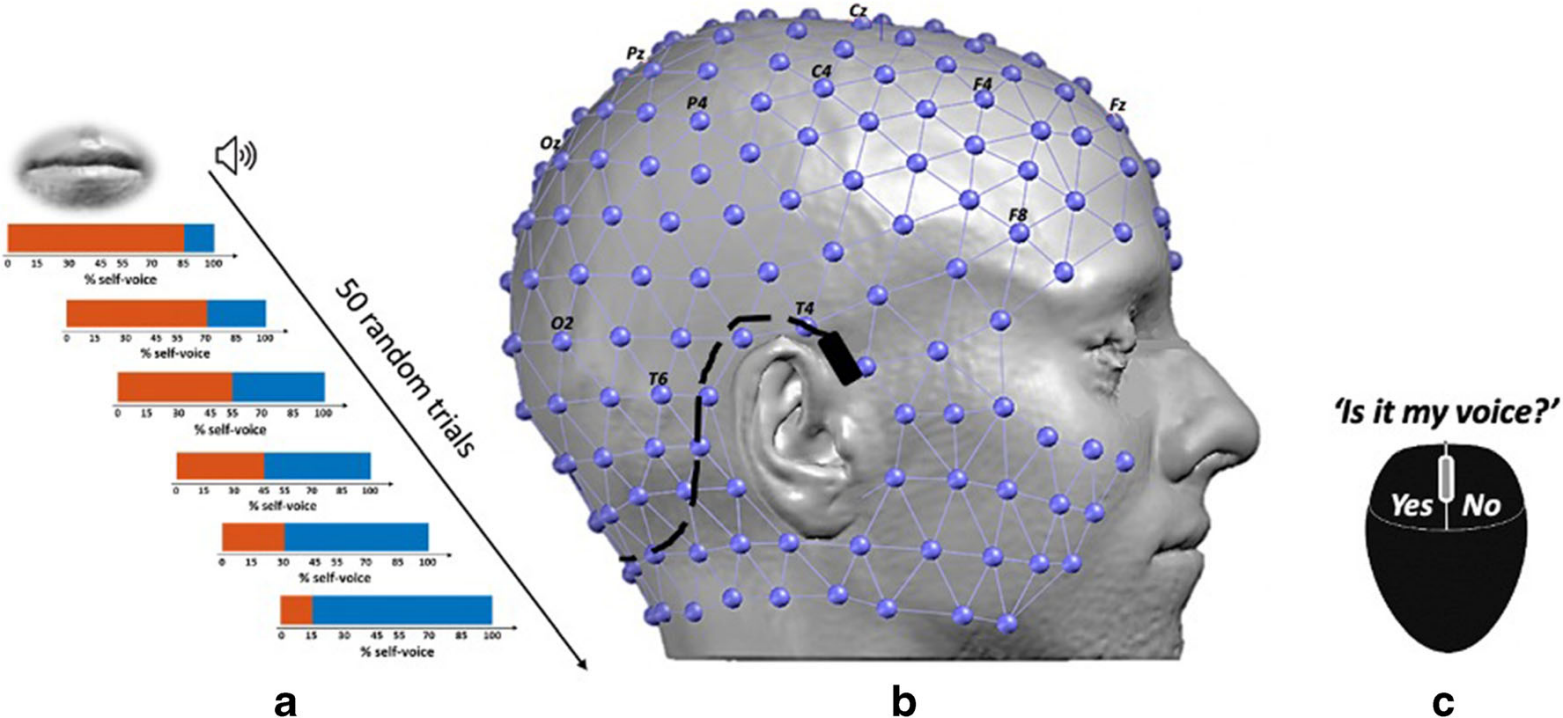
Self-other voice discrimination task: Each participant’s voice was recorded prior to the experiment while vocalizing phoneme /a/ for 2 s. Participant’s voice (the orange bar in **a**) was than morphed with a voice of a gender-matched unfamiliar person (the blue bar in **a**) in order to generate a self-other voice identity continuum. From that continuum, six voice morphs (% self-voice 15, 30, 45, 55, 70, 85) were presented randomly to participants while recording the electrophysiological activity with a high-density EEG cap (lilac spheres and connections in **b**). The cap is formed by 256 electrodes organized as an extension of the standard clinical 10–20 setup (whose electrodes names are indicated in black in **b**). After hearing a voice morph, participants were asked to indicate whether the voice they heard sounded more like their own or someone else’s by pressing the corresponding mouse button (**c**). Voice morphs were presented either through laptop loudspeakers (not illustrated) or a bone-conduction headset (illustrated in black in **b**)

**Fig. 2:**
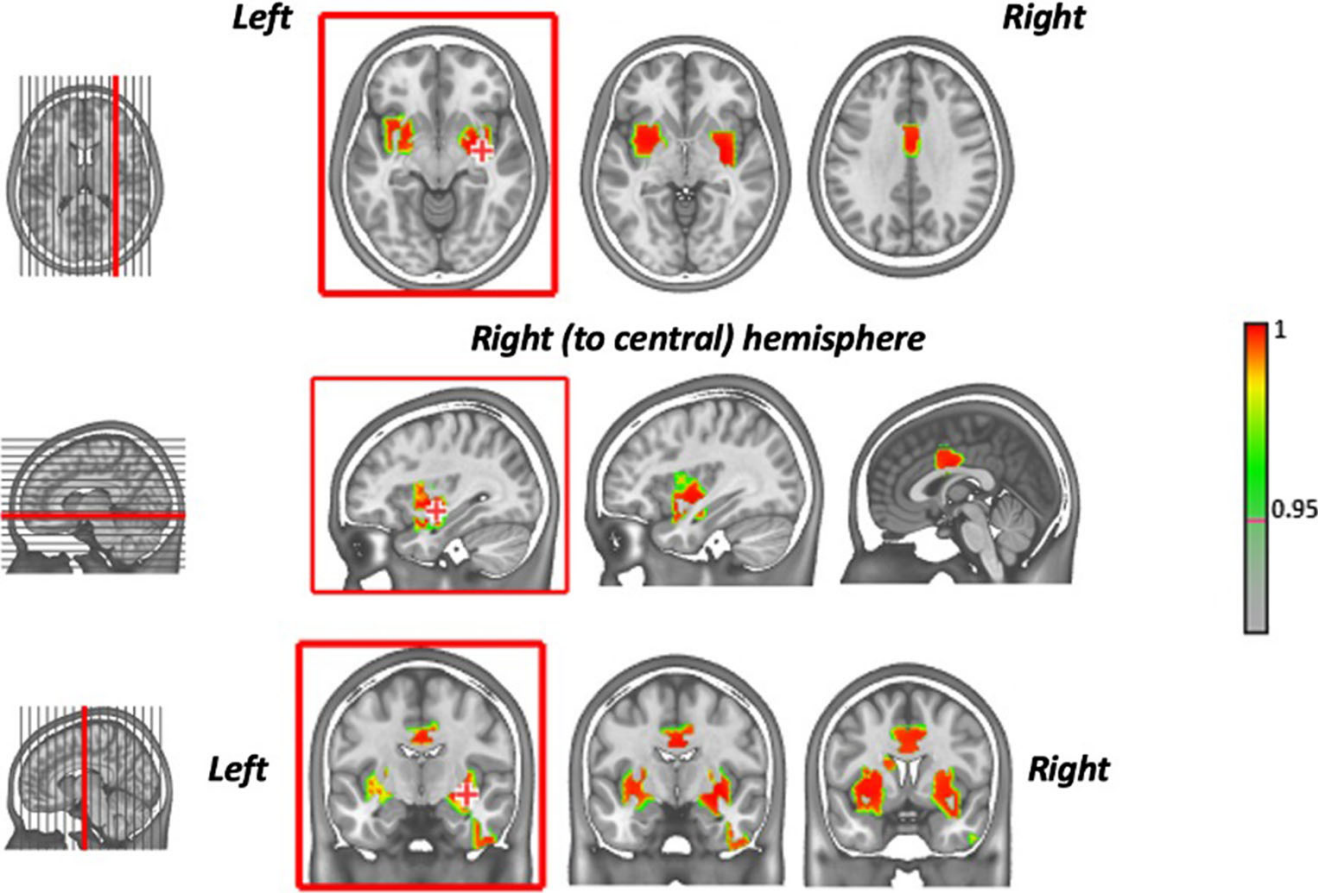
Localization of the self-voice: The analysis of the EEG during the voice task discrimination allows to define the network that significantly and specifically activates when participants hear their own voice. The results are visualized on a MNI template, and the results of activation have been obtained by projecting in the “brain space” (inverse space) the EEG signal acquired on the scalp, with the academic free Cartool software (https://sites.google.com/site/cartoolcommunity/). The brain network of the self-voice includes the insulae and putamen and the maximum of activation is lateralized on the right hemisphere (red crosses on the brain images in the red boxes). Moreover, the network includes the middle cingulum and part of the right inferior temporal pole [from Orepic et al., in submission]

Recognizing our own voice as being our own is an essential feature contributing to the constitution of our own self. That applies to internal listening as well as to the spoken voice. Auditory testing by the use of dedicated earphones, based on bone-conduction, includes the presentation of a sentence or vocalization of the vocal /a/—once by the test person’s own voice, and once by someone else’s voice. Furthermore, artificially generated voice morphs allow a stepwise mix (i.e., 90%/10% or 30%/70) between the two voices [67] (Fig. 1). Then, the threshold for recognition of one’s own voice may be defined. The addition of structural brain imaging and co-registered high-resolution EEG to that auditory testing paradigm allows to examine the various underlying neural activation patterns, which are different for self-voice and other voices (Fig. 2) [32, 54]. Interesting advancements have been made towards elucidating the etiology of auditory verbal hallucinations (AVH) in schizophrenia [85, 86]. The authors used a robotic procedure able to engender mild hallucinations in healthy individuals [19] to experimentally induce specific misattributions of self-towards-other voices [86], thereby mimicking the AVH phenomenology [48, 49]. Interestingly, such robotically induced self-voice misperceptions were further related to breathing [85], demonstrating a relationship between self-voice perception, interoception, and sensorimotor integration.

Another type of external stimuli, which are part of the concept of multisensory integration of interoceptive and exteroceptive stimuli, are those of visual origin. Interestingly, visual stimuli, which are presented synchronously with the frequency of the individual’s heartbeat, do not reach the threshold of awareness [106]. Those visual stimuli, however, which are presented asynchronously are reaching awareness. That cardio-visual suppression effect could be shown in a series of ECG-controlled visual attention experiments in conjunction with simultaneous 7T fMRI. Here, again, the insular cortex has been confirmed to play a crucial role in filtering and submitting intero- and exteroceptive signals to a hierarchy, which is related to self-awareness [106, 130].

The combination of the above tests with high-resolution EEG source imaging (ESI), co-registered to structural MRI, is very precise for functional anatomical allocation [76]. It could be shown to reliably identify the central sulcus with ESI of somatosensory evoked potentials [70]. The experimental setup to achieve such high spatio-anatomical resolution is based on electrical source imaging (ESI) of high-density (EEG) recordings (256 channels) using individual head models and distributed inverse solutions [75].

## Heartbeat evoked potentials (HEPs)

**Fig. 3:**
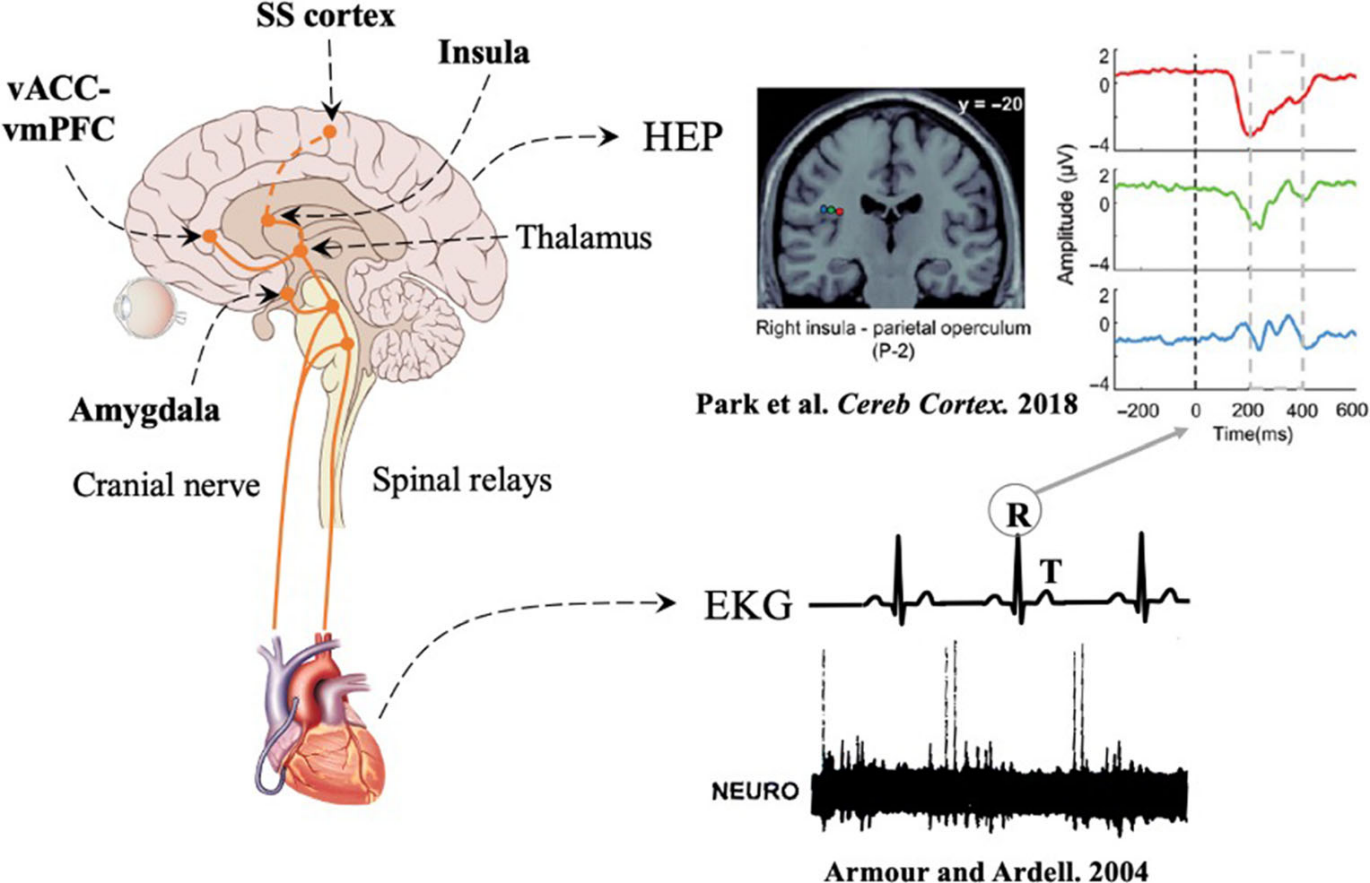
Heartbeat evoked potentials (HEPs). Mechanoreceptors on the heart wall discharge at a specific phase of EKG. Visceral information is then relayed up through cranial nerves and spinal relays to cortical and deep structures, among which the amygdala, the region of the ventral anterior cingulate cortex-ventral anterior prefrontal cortex (vACC-vmPFC), the insula, and the somatosensory cortex (SS cortex) play an important role in the integration of these signals. HEP can then be recorded in this region just after the R-wave on the EKG (electrocardiogram). Park et al. *Cereb Cortex* [91], Armour and Ardell [5]

**Fig. 4:**
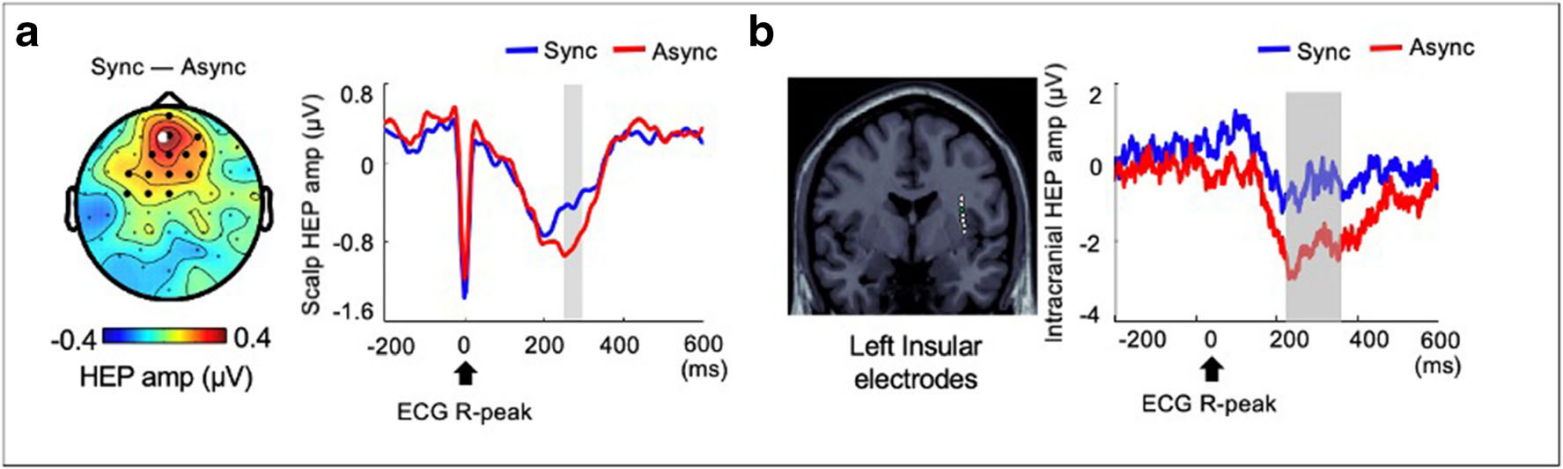
Self-related HEPs. **a** BSC modulations which were experimentally induced using synchronous or asynchronous visuo-tactile stimuli were associated with the HEP amplitude at the fronto-central region. A follow-up intracranial EEG study confirmed that self-related HEP can be measured at the insular cortex (**b**). HEP heartbeat evoked potential

**Fig. 5:**
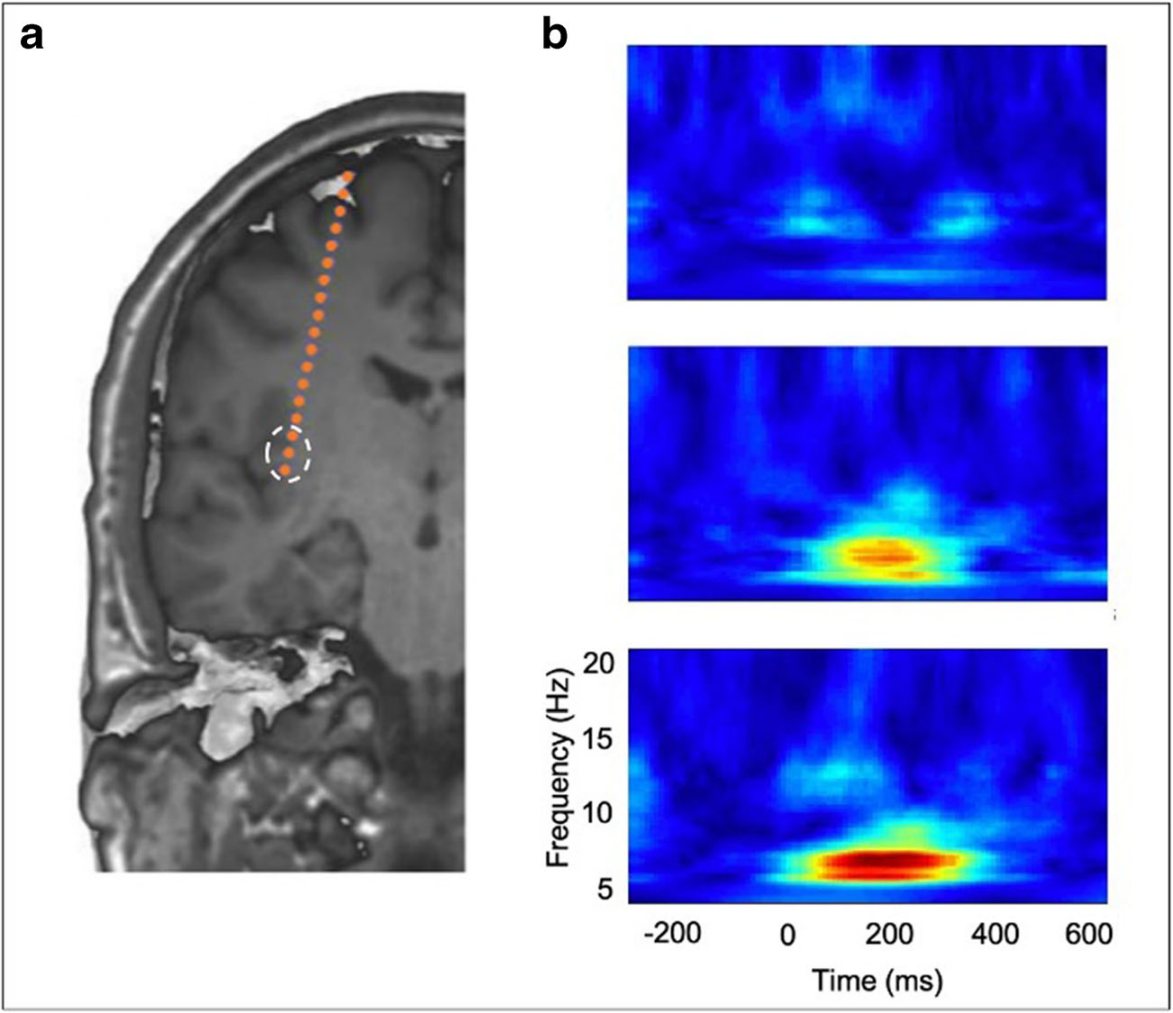
HEP during anesthesia. **a** During operation, intracranial EEG signals were recorded from contacts on the posterior insular cortex. **b** Spectral power of HEP from the posterior insular cortex. Increased power was observed around 200 ms after the ECG R-peak onset, from 5- to 10-Hz range. HEP heartbeat evoked potential

We think that especially HEPs are an important future measure of the sense of self in the pre-surgical evaluation or neurosurgical patients. HEPs—in analogy to MEPs or SEPs—are measurable brain activity related to muscular contractions of the heart (heartbeats). They reflect neural activity in cortical regions that process afferent cardiac signals within a time-window ranging from 200 to 650 ms following the R-peak [55, 68, 91] (Fig. 3). HEPs can be measured by high-resolution scalp EEG or by direct recording from intracranial electrodes [90, 91]. It is of importance to record averaged neural activity time-locked to ECG in order to identify the HEPs consistently. In a series of patients [91], who underwent implantation of SEEG electrodes (total, 599 intracranial contacts) for pre-surgical evaluation of intractable epilepsy, psychophysiological testing (neuropsychological exams, FBI test with head-mounted VR display as described above) was performed. Two principal anatomical regions, as the probable primary sources for HEPs, were found: the operculum and the insula. Experimentally induced alterations of self-identification were reflected by insular HEP pattern (Figs. 4 and 5). In MEG studies, recording of neural responses to cardiac signals in the so-called default network and in the insula was related to cognitive self involving spontaneous thoughts [9]. Thus, the amplitude of neural responses to heartbeats in the vmPFC, posterior cingulate cortex (PCC), and insula has been shown to be associated with the cognitive self (i.e., self-relatedness of spontaneous thought fluctuations) [9–11]. It is also important to mention that the insula—especially its anterior part together with the anterior and middle cingulate cortices (ACC/MCC)—is playing a crucial role in salience attribution. In fact, as part of the “salience network” [113], the anterior insula is hypothesized to detect the most relevant bottom-up events among internal and external environments and to temporarily initiate attentional control inputs, which then are sustained by the ACC [74]. Moreover, the right anterior insula is involved in switching between the (externally oriented) central-executive and the (self-referential) default-mode networks [122]. Taking together, the insular cortex may integrate visceral afferent signals, external visual and auditory signals, and saliency-related signals, allowing the mobilization of sustained attention, also via the involvement of other regions. We speculate that HEPs in association with tasks associated with the bodily self and the cognitive self could be used as biomarkers to determine regions that if resected would lead to post-surgical alterations of the sense of self. Preliminary results obtained under general anesthesia are promising.

## Processing of audio-tactile and peripersonal space (PPS)

The PPS is the space around a person, where direct human interaction with our environment takes place [56, 62]. To precisely allocate, i.e., audio-tactile stimuli in time and space, intactness of (unconscious) multisensory integration at the neural level is required [107]. Multisensory integration has been shown necessary to create and to maintain an intact PPS and BSC [20]. In a series of experiments in epilepsy patients with intracranial electrodes, such multisensory PPS responses were detected at distinct times after stimulus onset, which consisted of robotic-controlled application of a vibration stimulus to the chest of the patients. Separately, or simultaneously, white noise was presented via earphones, simulating to be approaching from the front [13]. Multisensory processing of auditive, tactile, and audio-tactile stimuli, respectively, involves the postcentral gyrus, the superior temporal gyrus, and the insula [13]. A recent fMRI study showed that spontaneous BOLD activity in the anterior and middle cingulate cortex (but also in the AI) can predict ascription of self-relatedness to the forthcoming noisy auditory stimuli [100]. An intra-operative feasibility study concerning PPS using an audio-tactile paradigm was recently conducted (J. Haemmerli et al., M.D. Thesis, still under production at the time of submitting this manuscript). Electrocorticography electrodes were placed on the two main regions of interest (i.e., parieto-temporal junction, fronto-basal region) during craniotomies and brain tumor removals. This primary study in anesthetized patients confirmed the feasibility to test PPS and multimodal integration on patients under narcosis with an intracranial recording.

## A plea for extra- and intra-operative recording of functions underlying the sense of self

The methods described above for extensive psychophysiological testing of the sense of self and related aspects of personality have proved to be consistently and safely applicable in healthy volunteers and in neurosurgical patients—extra-operatively so far. Here, we propose to develop similar test paradigms for the application during intracranial surgery of i.e. paralimbic gliomas affecting the insula, or during epilepsy surgery. As the full test battery, including audio-tactile and visual testing combined with HEP recording may take more than 2 h, testing will need to be shortened and optimized to the surgical setting. We also note that elements of the above tests may become a routine part of the pre-surgical investigation of epilepsy patients, in whom SEEG electrodes or subdural grid electrodes were placed already, or during DBS procedures. Even if not all possible psychophysiological tests are performed, a more widespread use will contribute to the ongoing efforts to decipher the underlying mechanisms of the sense of self.

Intra-operatively, the portfolio of neuropsychological tests during awake craniotomy may be extended by some of the described test, i.e., by the addition of head-holder-mounted VR displays and the simultaneous recording of HEPs. HEPs on the other hand, once proved that stable intra-operative recording is feasible, even under general anesthesia, may serve as a biomarker for the capacity of multisensory integration as a surrogate for intactness of cortical cardiac processing and related aspects of BSC in patients undergoing brain surgery. Concurrently to these recent developments in psychophysics, new generations of ultra-thin and very flexible electrodes are under development and preclinical evaluation [111]. These visco-elastic electrodes, which have micro-electronical properties beyond simple recording, may be more easily placed on irregular cortical surface patterns than this is the case with traditional subdural strip and grid electrodes [112]. They interfere lesser with the course of surgery and thus will further ignite the ambition to broaden the perspectives of intra-operative surveillance during intracranial procedures [112].

## Conclusions and outlook

Technical advances have always strongly impacted neurosurgical practice, especially over the past few decades: neuronavigation, intra-operative imaging and fluorescence, DBS, and the widespread acceptance of intra-operative neuromonitoring. All these tools or techniques require investment on the financial as well as on the personal side. Yet, their obvious clinical benefits outweigh the related expenses, at least in health care systems, where there is sufficient funding to pay for technological progress and surgical evolution. Whereas there is widespread acceptance to integrate cognitive neuroscience and neuropsychology procedures in the pre-operative evaluation and in the post-operative follow-up of i.e. neuro-oncological patients, intra-operative neuromonitoring has long been limited to functions, such as movement during i.e. glioma surgery in the central region and i.e. CN VII and BAEP monitoring for surgery in and around the cerebello-pontine angle. The revival of awake craniotomy has brought along a surge of techniques for intra-operative awake mapping of white matter fiber tracts, i.e., for the protection of language and speech. Nowadays, olfactory monitoring, VEPs, and integrated suction-resection mapping devices complete the armamentarium for monitoring and mapping of precisely allocated functions.

Nevertheless, the present state of technology and the neuroscience of interoceptive and of exteroceptive aspects of the sense of self, as well as cognitive aspects of the self, allow to go one step further: Based on the above concepts and experimental paradigms, it is possible to test fully cooperative patients with i.e. low-grade gliomas, or with lesions in the frontal, the paracentral, the parietal, the temporal, and the insular lobes in the sense of self as based on auditory, visual, visuo-tactile, and interoceptive stimuli. Alterations of these brain functions may be followed by changes in the bodily self, the cognitive self, or other related aspects (i.e., personality). These pre- and post-operative behavioral, neural, and neuropsychological assessments require dedicated hardware and reseach staff. It is entirely feasible to conduct similar tests during awake craniotomy by the use of i.e. head-holder-mounted VR displays and by the use of newly developed ultra-thin cortical electrodes, which may follow the irregular geometry of the various brain regions. HEPs as biomarkers for the interoceptive component of the sense of self may be recorded and analyzed under the conditions of awake craniotomy and of general anesthesia. The role and the impact of that knowledge on patient counseling (i.e., the intra-operative decision to go for a more extensive tumor resection or not, if the risk to damage zones of multisensory integration is implied) has yet to be clarified. Systematically mapping psychophysiological alterations might however allow to quantify the risk of post-surgical perturbation of the sense of self.

Nowadays, intra-operative BSC monitoring is difficult to set-up in terms of time spent during the surgery and in terms of financial issue. BSC monitoring is, however and at least in part, technically feasible. Intra-operative electrophysiology setups have already most of these technical solutions. These setups have already been applied in different research domains (epilepsy, deep brain stimulation, vision, speech, olfaction, all under general anesthesia). Beyond clinical systems, they include several key elements: high-frequency electrophysiological brain signal recordings, captured from any kind of recording electrodes; internal or external open trigger sources for trigged recordings, diverse signal processing analyses including online analyses. Dedicated engineers have this particular expertise and experience to manage intra-operatively these setups and to analyze these signals. Intra-operative BSC monitoring represents also a potential risk for neurosurgical patients because of the additional time during which patients are anesthetized. Only a strict intra-operative coordination between neurosurgeons, electrophysiologists, and anesthesiologists can reduce the risk. Furthermore, the use of intra-operative MRI in the BSC monitoring will play a key role in recognition of the targeted brain areas. The whole intra-operative neuromonitoring setup should fit to intra-operative MR prerogatives. One main limitation has also to be highlighted, in that BSC monitoring requires specific technological instruments, which are not ubiquitously available even in modern neurosurgical centers.

Numerous open questions remain, of course, as many neuroimaging studies related to the self are correlative in nature. Thus, the causal role of the supposedly involved brain areas (e.g., insula and temporal-parietal junction) of the bodily self or cognitive self has to be determined yet. Then, the interactions or overlap between brain networks underlying these two notions of self is also unknown. Finally, it is not known whether bodily signals and their neural representations only impact the bodily self or whether they also modulate cortical networks of the cognitive self, impacting aspects of the self-related to personality traits. But joint efforts between neurosurgeons, clinical neuroscientists, engineers, and experts in signal analysis—inside and outside the operating room—will help allow to advance understanding of the human self and reduce unwarranted neurosurgical side effects.
